# Cancer-Associated Fibroblast Heterogeneity Shapes Prognosis and Immune Landscapes in Head and Neck Squamous Cell Carcinoma

**DOI:** 10.3390/cancers18020215

**Published:** 2026-01-09

**Authors:** Hideyuki Takahashi, Hiroyuki Hagiwara, Hiroe Tada, Miho Uchida, Toshiyuki Matsuyama, Kazuaki Chikamatsu

**Affiliations:** Department of Otolaryngology-Head and Neck Surgery, Graduate School of Medicine, Gunma University, 3-39-22 Showa-machi, Maebashi 371-8511, Gunma, Japantikamatu@gunma-u.ac.jp (K.C.)

**Keywords:** head and neck squamous cell carcinoma, cancer-associated fibroblasts, tumor microenvironment, single-cell RNA sequencing, inflammatory fibroblasts, prognostic biomarkers, immune landscape, recursive partitioning analysis

## Abstract

**Background/Objectives:** Head and neck squamous cell carcinoma (HNSCC) is a biologically heterogeneous malignancy with poor outcomes in advanced disease. Increasing evidence indicates that the tumor microenvironment, particularly cancer-associated fibroblasts (CAFs), plays an important role in tumor progression and immune regulation. However, the diversity of CAF subsets and their clinical relevance in HNSCC remain incompletely understood. This study aimed to characterize CAF heterogeneity and assess the prognostic significance of CAF subset-specific transcriptional programs. **Methods:** Single-cell RNA sequencing data from HNSCC tumors were analyzed to identify CAF subsets based on differentially expressed genes. CAF subset-specific gene signatures were used to construct prognostic risk models for overall survival (OS) and progression-free survival (PFS) in The Cancer Genome Atlas HNSCC cohort, with validation in an independent dataset. CAF-driven prognostic groups were defined, and their immune landscapes and biological pathways were evaluated. Bulk RNA sequencing of primary CAF cultures was performed for validation. **Results:** Six CAF subsets were identified, including myofibroblastic (myCAF), inflammatory (iCAF), antigen-presenting, and extracellular matrix-related CAFs. Risk scores derived from inflammatory CAF subsets consistently predicted shorter OS across independent cohorts, whereas PFS prediction showed greater cohort dependency. CAF-based stratification identified patient subgroups with distinct immune profiles and pathway enrichment patterns. These results were supported by validation analyses and by bulk RNA sequencing of primary CAFs, demonstrating preservation of myCAF- and iCAF-like transcriptional programs ex vivo. **Conclusions:** CAF heterogeneity has important prognostic and immunological implications in HNSCC. Inflammatory CAF-related transcriptional programs represent robust markers of patient survival and may complement tumor-intrinsic biomarkers.

## Simple Summary

Cancer progression is influenced not only by cancer cells themselves but also by surrounding supportive cells in the tumor environment. Among these, cancer-associated fibroblasts play important roles in shaping tumor behavior and immune responses, but their diversity and clinical significance in head and neck cancer are not fully understood. In this study, we analyzed single-cell gene expression data to identify distinct fibroblast subtypes in head and neck squamous cell carcinoma. We further examined whether gene signatures derived from these fibroblast subtypes could predict patient outcomes using large clinical datasets. We found that specific inflammatory fibroblast programs were strongly associated with patient survival and distinct immune environments. Importantly, these fibroblast-related patterns were reproducible across independent patient cohorts and were supported by additional experiments using cultured fibroblasts. Our findings provide new insights into the biological and clinical relevance of fibroblast diversity and suggest that stromal features may complement existing biomarkers to improve risk stratification in head and neck cancer.

## 1. Introduction

Head and neck squamous cell carcinoma (HNSCC) is a biologically heterogeneous malignancy characterized by diverse clinical behaviors and treatment responses [1,2]. Despite advances in surgery, radiotherapy, and systemic therapies, including immune checkpoint inhibitors, the prognosis of patients with advanced HNSCC remains poor [1,3,4,5]. Increasing evidence indicates that tumor progression and therapeutic resistance are strongly influenced not only by malignant epithelial cells but also by the tumor microenvironment (TME), particularly the stromal compartment [6,7,8].

Cancer-associated fibroblasts (CAFs) represent one of the most abundant and functionally diverse stromal cell populations within the TME [9,10,11]. CAFs contribute to tumor growth, invasion, immune modulation, and therapeutic resistance through extracellular matrix remodeling, cytokine and chemokine secretion, and direct interactions with immune cells [11,12,13,14,15]. Recent single-cell RNA sequencing (scRNA-seq) studies have revealed substantial heterogeneity among CAFs, leading to the identification of distinct functional subsets, including myofibroblastic CAFs (myCAFs), inflammatory CAFs (iCAFs), and antigen-presenting CAFs (apCAFs) [10,16,17,18]. However, the composition, functional diversity, and clinical relevance of CAF subsets in HNSCC remain incompletely understood.

Several studies have suggested that specific CAF phenotypes are associated with tumor aggressiveness and patient outcomes [10,11,19]. In particular, inflammatory CAF programs characterized by cytokine and chemokine expression have been implicated in immune suppression and tumor-promoting inflammation [20,21]. Nevertheless, most prior investigations have focused on CAF heterogeneity at the single-cell level without systematically integrating these findings with large-scale clinical outcome data. Moreover, whether CAF subset-specific transcriptional programs can be translated into robust prognostic models applicable across independent cohorts has not been fully addressed.

In this study, we comprehensively characterized CAF heterogeneity in HNSCC using scRNA-seq data and identified six distinct CAF subsets with unique transcriptional features. By integrating CAF subset-specific gene signatures with bulk transcriptomic data from The Cancer Genome Atlas (TCGA) and an independent validation cohort, we developed and validated prognostic models for overall survival (OS) and progression-free survival (PFS). Furthermore, we applied recursive partitioning analysis to define CAF-driven prognostic subgroups and investigated their associated immune landscapes and biological pathways. Finally, we validated key CAF transcriptional programs using bulk RNA sequencing of primary CAFs derived from HNSCC tissues. Together, our findings provide a comprehensive framework linking CAF heterogeneity, immune contexture, and clinical outcomes in HNSCC.

## 2. Materials and Methods

### 2.1. Acquisition of the GSE164690 Dataset

The GSE164690 dataset was obtained from the Gene Expression Omnibus (GEO) database (http://www.ncbi.nlm.nih.gov/geo/). This publicly available scRNA-seq dataset was generated from freshly resected primary HNSCC tumors, comprising both HPV-positive and HPV-negative cases. In this dataset, CD45-negative cells were isolated prior to sequencing, enabling focused analysis of non-hematopoietic stromal and epithelial compartments, including fibroblasts. CD45-negative cells were analyzed using the Seurat R package (version 4).

### 2.2. scRNA-Seq Data Processing

Cells expressing fewer than 100 genes were excluded. Global-scaling normalization was performed using a scale factor of 10,000. A total of 2000 highly variable features were selected for downstream analyses. Nonlinear dimensional reduction was performed using Uniform Manifold Approximation and Projection (UMAP). Cell clustering was conducted using the FindClusters function, and cell identities were annotated based on differentially expressed genes (DEGs) identified using the FindAllMarkers function.

### 2.3. Development of CAF Subset-Related Survival Prediction Models

Using DEGs for each CAF subset (adjusted *p* < 0.01), we developed subtype-specific survival prediction models for HNSCC. RNA sequencing data (Illumina HiSeq RNAseq V2 normalized counts) and clinical information for 520 patients in TCGA were obtained from FireBrowse (http://firebrowse.org/), including 97 HPV-positive and 423 HPV-negative cases. Univariate Cox proportional hazards regression was used to evaluate associations with OS and PFS. Genes with *p* < 0.05 were selected for further analysis. Least absolute shrinkage and selection operator (LASSO) regression was performed using the glmnet R package (version 4.1.8) to identify optimal predictive features, with 10-fold cross-validation to determine the optimal lambda (λ). Risk scores were calculated as follows: risk score = exp Σ[coefficient × (gene expression − mean gene expression)]. Risk groups were compared using Kaplan–Meier analysis and the log-rank test. Optimal risk cutoffs were determined using receiver operating characteristic curves. Multivariate Cox regression was performed for variables with *p* < 0.05 in univariate analyses. Recursive partitioning analysis (RPA) for survival modeling was conducted using the partykit R package (version 1.2.24).

### 2.4. Gene Set Enrichment Analysis (GSEA)

GSEA (v4, Broad Institute, Cambridge, MA, USA) was performed to identify pathways upregulated in terminal nodes obtained from RPA. Normalized enrichment score (NES), *p*-value, and false discovery rate (FDR) q-value were calculated using the Hallmark gene sets.

### 2.5. Deconvolution Analysis

CIBERSORTx (Stanford University, Stanford, CA, USA) was used to estimate immune cell fractions in terminal nodes. The LM22 signature matrix was used to calculate infiltration scores for 22 immune cell types. Heatmaps were generated using the pheatmap R package (version 1.0.13).

### 2.6. Validation of Risk Scores and Survival Models

For external validation, we analyzed the GSE65858 dataset, which includes microarray data (Illumina HumanHT-12 v4.0) and clinical information for 270 patients (73 HPV-positive, 196 HPV-negative, and 1 HPV-unknown). Risk scores were calculated using the coefficients derived from the TCGA cohort. Patients were stratified into four terminal nodes based on RPA grouping. GSEA and CIBERSORTx analyses were performed as described above.

### 2.7. Isolation and Culture of CAFs

Tumor specimens from 10 newly diagnosed HNSCC patients (five oropharyngeal and five hypopharyngeal cancers) were obtained. CAFs were isolated as previously described [12,13]. Briefly, tissues were minced (1–3 mm^3^), treated with antibiotics, and cultured in DMEM supplemented with 10% fetal bovine serum, 2 mM L-glutamine, and antibiotics. After several passages, fibroblasts were characterized by flow cytometry for fibroblast activation protein (FAP), CD90, and α-smooth muscle actin (α-SMA). All CAFs were used before passage 10. Institutional Review Board approval (Gunma University, No. HS2017-152) and written informed consent were obtained.

### 2.8. RNA Sequencing of CAFs

Total RNA was extracted using the FastGene RNA Premium Kit (Nippon Genetics, Tokyo, Japan). RNA integrity was confirmed using Agilent Bioanalyzer (RIN > 9.7). Libraries were prepared using the KAPA mRNA HyperPrep Kit (Kapa Biosystems Inc., Wilmington, MA, USA) and SeqCap adapters (Roche Sequencing Solutions, Pleasanton, CA, USA), followed by paired-end sequencing (43 bp) on the Illumina NextSeq500 platform (Illumina, San Diego, CA, USA). Reads were aligned to the hg19 reference genome using STAR (v2.5.3a), and quantification was performed using RSEM (v1.3.3). Normalized log_2_ expression values were calculated, and samples were grouped using unsupervised hierarchical clustering based on CAF subset-specific DEG z-scores. Heatmaps were generated using pheatmap.

### 2.9. Statistical Analysis

All statistical analyses were performed using R (version 4.3.1). One-way ANOVA with Tukey’s post hoc test was used for continuous variables, and chi-square tests were used for categorical variables. Two-sided *p* < 0.05 was considered statistically significant.

## 3. Results

### 3.1. Single-Cell Transcriptomic Profiling Reveals Six Distinct CAF Subsets in HNSCC

**Figure 1:**
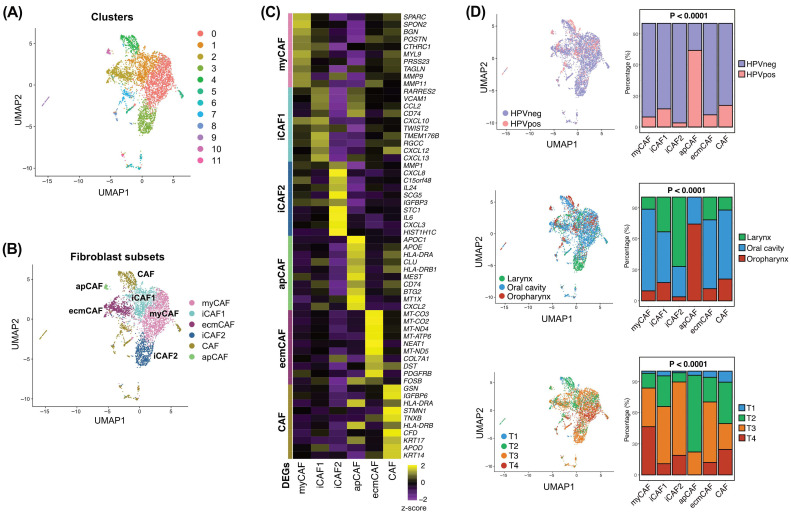
Identification and characterization of CAF subsets in HNSCC. (**A**) UMAP plot of fibroblasts isolated from CD45^−^ cells showing 12 transcriptionally distinct clusters. (**B**) UMAP plot of six CAF subsets with their relative proportions within the fibroblast population: myCAF (32.5%), iCAF1 (19.2%), iCAF2 (14.0%), apCAF (1.1%), ecmCAF (14.1%), and merged other CAFs (CAF; 19.1%). (**C**) Heatmap showing the top 10 differentially expressed genes for each CAF subset. (**D**) Proportions of CAF subsets stratified by HPV status, anatomical site, and T stage. CAF, cancer-associated fibroblast; HNSCC, head and neck squamous cell carcinoma; UMAP, Uniform Manifold Approximation and Projection; HPV, human papillomavirus.

We first characterized the fibroblast populations within the tumor microenvironment using scRNA-seq data. Cells were then clustered into six different cell types (epithelial cell, endothelial cell, fibroblast, lymphoid cell, myeloid cell, and pericyte) as previously described [22]. Subsequently, 4409 fibroblasts were extracted and clustered into 12 fibroblast clusters (Figure 1A). Based on the DEGs in each cluster, six CAF subsets were finally identified, including myCAF, iCAF1, iCAF2, ecmCAF, apCAF, and merged other CAFs (CAF) (Figure 1B,C). myCAFs exhibited high expression of extracellular matrix (ECM)-related genes, including *SPARC*, *SPON2*, *POSTN*, *TAGLN*, and *MMP11*, consistent with a contractile and matrix-producing phenotype. Two inflammatory CAF subsets were identified, each defined by a unique transcriptional signature. iCAF1 exhibited high expression of chemokines (*CCL2*, *CXCL10*, *CXCL12*, and *CXCL13*) and immune-modulatory genes (*VCAM1* and *CD74*), suggesting a role in recruiting and regulating immune cells. In contrast, iCAF2 expressed elevated levels of inflammatory cytokines and chemokines (*IL6*, *IL24*, *CXCL8*, and *CXCL3*) together with *MMP1*, indicating a strongly inflammatory and matrix-remodeling phenotype distinct from iCAF1. ecmCAFs were enriched for ECM structural and remodeling genes such as *COL7A1*, *TNXB*, and *PDGFRB*, suggesting a role in matrix organization. apCAFs showed elevated expression of antigen presentation-related genes, including *HLA-DRA*, *HLA-DRB1*, and *CD74*, which is consistent with previously described antigen-presenting fibroblasts. The merged CAF cluster was characterized by expression of epithelial and stress-associated genes such as *KRT14*, *KRT17*, and *FOSB*, indicating a distinct reactive stromal state.

To assess clinical relevance, we examined the distribution of CAF subsets across clinical variables (Figure 1D). apCAFs were significantly enriched in HPV-positive oropharyngeal tumors, and they also appeared at a higher proportion in early-stage cancers (T1–T2) compared with advanced tumors. This pattern suggests that apCAFs may emerge preferentially in earlier tumor evolution, potentially reflecting a stromal state associated with antigen presentation or immune activation. The proportions of the remaining CAF subsets varied significantly across anatomical subsites and T stages (all *p* < 0.0001), indicating that CAF heterogeneity is associated with tumor location and disease progression.

### 3.2. Construction of CAF Subset-Specific Prognostic Models Using LASSO Regression

**Figure 2:**
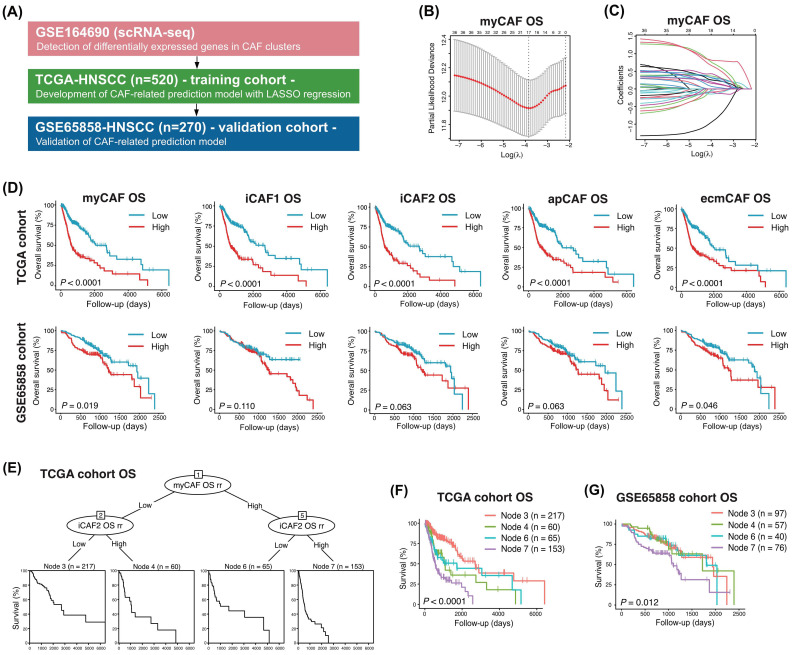
Construction of CAF subset-specific prognostic models and CAF-driven risk stratification. (**A**) Overview of the analytical workflow for constructing CAF subset-specific prognostic models using scRNA-seq-derived gene signatures and bulk transcriptomic data. (**B**,**C**) LASSO regression analyses for overall survival (OS) based on DEGs from CAF clusters, with representative coefficient profiles and optimal lambda selection shown for the myCAF subset. (**B**) Cross-validation plot showing the mean cross-validated partial likelihood deviance (red points) at each value of λ, with error bars indicating ±1 standard error. (**C**) LASSO coefficient paths for all candidate genes. Each colored line represents the trajectory of a gene-specific Cox regression coefficient across values of log(λ), shrinking toward zero as penalization increases. (**D**) Kaplan–Meier survival curves for OS stratified by CAF subset-specific risk scores in the TCGA and GSE65858 cohorts. (**E**) Conditional inference tree generated by recursive partitioning analysis integrating CAF risk scores in the TCGA cohort. (**F**) Kaplan–Meier curves for OS across terminal nodes identified in the TCGA cohort. (**G**) Validation of OS-based terminal nodes in the GSE65858 cohort. LASSO, least absolute shrinkage and selection operator.

We constructed prognostic prediction models based on DEGs of each CAF subset using the TCGA cohort and validated them in the GSE65858 cohort (Figure 2A). To evaluate the prognostic significance of CAF subsets, DEGs associated with OS and PFS were first identified by univariate Cox regression in TCGA-HNSCC (n = 520). LASSO regression was then applied to derive CAF subset-specific risk signatures (Figure 2B,C, Supplementary Figure S1). In the TCGA cohort, CAF risk scores significantly stratified patients into high- and low-risk groups for OS across all CAF subsets (Figure 2D). In the GSE65858 cohort, similar stratification trends were observed for several CAF subsets, although statistical significance was not consistently achieved across all subsets (Figure 2D). For PFS, Kaplan–Meier curves demonstrated comparable stratification patterns in both TCGA and GSE65858 cohorts; however, these differences did not reach statistical significance in the validation cohort (Supplementary Figure S2A). Clinical correlates of the CAF risk scores were further examined (Table 1 and Supplementary Table S1). Higher CAF risk scores—particularly those derived from iCAF1 and iCAF2—were significantly associated with adverse clinical features, including advanced T stage and HPV-negative status. Similar associations were observed when PFS-related risk scores were evaluated, reinforcing the linkage between inflammatory CAF programs and aggressive tumor behavior. In multivariate Cox regression, iCAF1 and iCAF2 risk scores consistently emerged as independent predictors of shorter OS and PFS in the TCGA cohort (Table 2). These findings indicate that inflammatory CAF programs have strong and clinically meaningful prognostic relevance in HNSCC, supported by both survival modeling and their correlation with established risk factors.

### 3.3. Recursive Partitioning Identifies CAF-Driven Patient Subgroups with Distinct Prognosis

Recursive partitioning analysis (RPA) integrating risk scores from multiple CAF subsets produced a conditional inference tree that stratified TCGA patients into four terminal nodes (Figure 2E). Node 3 represented a low-risk group with the most favorable OS, while Node 7 represented the highest-risk group. Kaplan–Meier curves confirmed significant OS differences among terminal nodes in the TCGA cohort (Figure 2F). These OS-based survival groups were validated in the GSE65858 cohort (n = 270), where patients were again separated into four prognostically distinct nodes with similar patterns (Figure 2G), demonstrating the robustness of the CAF-based prognostic model.

For PFS, the RPA applied to the TCGA cohort also identified four terminal nodes that showed significant stratification (Supplementary Figure S2B). However, although the same RPA structure was reproduced in the GSE65858 cohort, Kaplan–Meier curves did not show statistically significant PFS differences among terminal nodes (Supplementary Figure S2C). These results suggest that while CAF-derived risk signatures are strong predictors of OS across datasets, their prognostic performance for PFS may be more cohort-dependent.

### 3.4. Immune Landscape and Biological Pathways Associated with CAF-Driven Prognostic Groups

**Figure 3:**
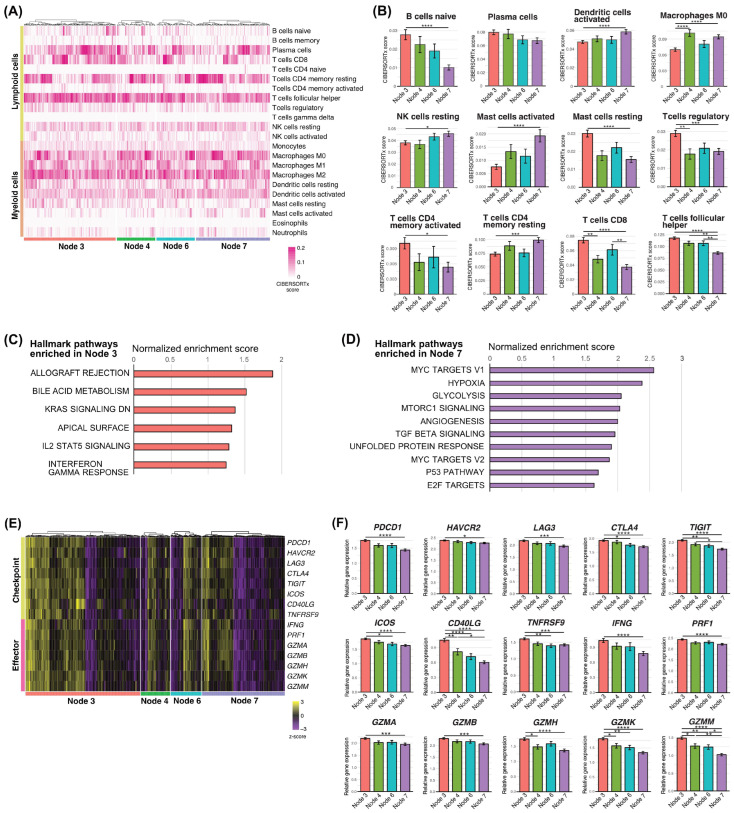
Immune landscape and biological pathways associated with CAF-driven prognostic groups. (**A**) Heatmap showing immune cell abundance estimated using CIBERSORTx in OS-based terminal nodes in the TCGA cohort. (**B**) Bar graphs displaying immune cell abundance scores in each terminal node. (**C,D**) Gene set enrichment analysis (GSEA) showing pathways enriched in Node 3 and Node 7. (**E**) Heatmap showing expression of immune effector and immune checkpoint-related genes across terminal nodes. (**F**) Bar graphs displaying relative expression levels of selected immune-related genes. *, *p* < 0.05; **, *p* < 0.01; ***, *p* < 0.001; ****, *p* < 0.0001.

Because CAF-derived risk signatures robustly predicted OS across datasets but showed limited reproducibility for PFS, we focused subsequent analyses on OS-based prognostic nodes. We then characterized the immune landscapes associated with these CAF-driven survival differences using CIBERSORTx. In the TCGA cohort, Node 7, which was associated with poor prognosis, showed higher infiltration of macrophages, activated mast cells, resting NK cells, resting CD4^+^ memory T cells, and activated dendritic cells. In contrast, Node 3, which exhibited the most favorable survival, was enriched for B cells, resting mast cells, regulatory T cells, activated CD4^+^ memory T cells, CD8^+^ T cells, and follicular helper T cells (Figure 3A,B). To assess the reproducibility of these immune patterns, we performed the same deconvolution analysis using the GSE65858 cohort. Despite differences in data platforms, similar trends were observed, with Node 3 consistently showing enrichment of lymphocyte populations associated with antitumor immunity, whereas Node 7 exhibited a myeloid- and inflammation-dominant immune profile (Supplementary Figure S3A,B).

Gene set enrichment analysis (GSEA) further supported these findings. In the TCGA cohort, Node 3 was significantly enriched for immune activation-related pathways, including allograft rejection, interferon gamma response, and IL2–STAT5 signaling, whereas Node 7 was enriched for pathways related to tumor progression and metabolic reprogramming, such as MYC targets, hypoxia, glycolysis, angiogenesis, and TGF-β signaling (Figure 3C,D). Comparable enrichment patterns were also observed in the GSE65858 cohort, reinforcing the biological distinction between favorable and unfavorable prognostic nodes (Supplementary Figure S3C,D).

Consistent with these pathway-level differences, expression levels of immune effector and immune checkpoint genes—including *IFNG*, *GZMB*, *PRF1*, *PDCD1*, *CTLA4*, *HAVCR2*, and *LAG3*—were significantly higher in Node 3 in the TCGA cohort, indicating a more active antitumor immune environment (Figure 3E,F). Similar expression trends were confirmed in the GSE65858 cohort (Supplementary Figure S3E,F), supporting the robustness of the CAF-driven immune stratification across independent datasets.

### 3.5. Validation of CAF Subset Signatures Using Bulk RNA-Seq of Primary CAFs

**Figure 4:**
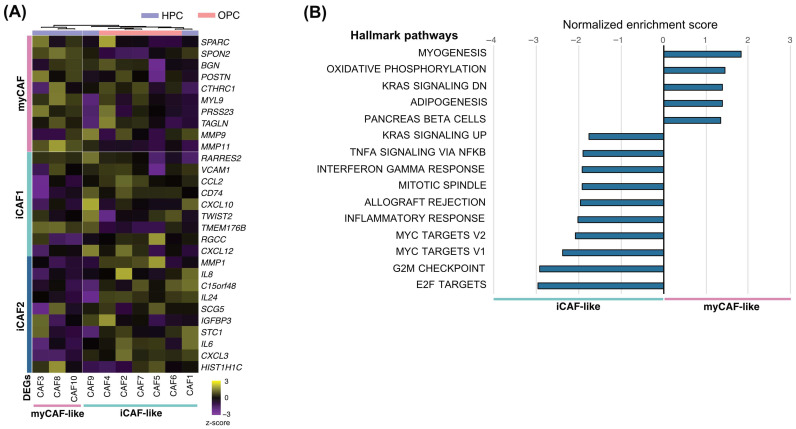
Validation of CAF transcriptional programs using bulk RNA sequencing of primary CAFs. (**A**) Heatmap showing unsupervised hierarchical clustering of primary CAF samples based on myCAF- and iCAF-specific DEGs. (**B**) GSEA showing pathways enriched in myCAF-like and iCAF-like CAFs derived from primary cultures. HPC, hypopharyngeal cancer; OPC, oropharyngeal cancer.

To further validate the transcriptional programs underlying myCAF and iCAF phenotypes, we performed bulk RNA sequencing of cultured CAFs established from freshly resected HNSCC tumors (n = 10). Given that myCAF-, iCAF1-, and iCAF2-related risk scores showed the strongest and most consistent associations with overall survival in the prognostic analyses, bulk RNA-seq-based validation focused on these CAF transcriptional programs. Hierarchical clustering of myCAF/iCAF-specific DEGs clearly separated samples into myCAF-like and iCAF-like groups (Figure 4A). Notably, all three myCAF-like samples were derived from hypopharyngeal cancers, whereas five of the seven iCAF-like samples originated from oropharyngeal cancers, suggesting potential anatomical site-associated differences in CAF transcriptional programs. GSEA revealed that iCAF-like CAFs were enriched for inflammatory pathways (inflammatory response, allograft rejection, interferon gamma response, and TNFα signaling via NFκB), whereas myCAF-like CAFs were enriched for ECM-related and proliferative pathways (myogenesis, oxidative phosphorylation, and KRAS signaling) (Figure 4B). These findings support the biological distinction between inflammatory and myofibroblastic CAF programs observed in scRNA-seq data.

## 4. Discussion

In this study, we integrated single-cell transcriptomics with large-scale clinical datasets to elucidate the prognostic relevance of cancer-associated fibroblast heterogeneity in HNSCC. Rather than focusing solely on CAF classification, our analyses demonstrate that inflammatory CAF programs constitute a central axis linking stromal biology, immune contexture, and patient survival.

A key finding of this study is the identification of two transcriptionally and functionally distinct inflammatory CAF subsets. While both iCAF1 and iCAF2 exhibited inflammatory signatures, their gene expression profiles suggested divergent roles within the tumor microenvironment. iCAF1 was characterized by chemokine-rich programs associated with immune cell recruitment and immune modulation, whereas iCAF2 displayed a cytokine- and matrix-remodeling-dominant profile, consistent with a more aggressive, tissue-destructive stromal phenotype. This subdivision of inflammatory CAFs extends previous CAF classifications and highlights functional heterogeneity within iCAFs that may have distinct biological and clinical consequences [16,17,20].

Importantly, CAF-derived risk signatures—particularly those from iCAF1 and iCAF2—emerged as robust predictors of overall survival across independent cohorts. These findings are consistent with accumulating evidence from HNSCC and other solid tumors indicating that distinct CAF phenotypes, especially inflammatory and immune-modulatory subsets, are closely associated with tumor aggressiveness and adverse patient outcomes [19,23,24,25,26]. The reproducibility of OS-based stratification suggests that CAF transcriptional programs capture stable features of tumor biology that are less sensitive to treatment-related variability. In contrast, the reduced reproducibility of progression-free survival stratification in the validation cohort likely reflects the multifactorial nature of disease progression, which may be influenced by treatment heterogeneity, surveillance intensity, and cohort-specific clinical practices. Together, these observations underscore overall survival as a more reliable endpoint for CAF-centered prognostic modeling in HNSCC. Moreover, the consistent stratification of overall survival observed across independent cohorts suggests that integrated CAF-derived risk signatures capture core stromal programs that remain informative despite substantial intertumoral heterogeneity in HNSCC.

Beyond prognostic stratification, our study provides insight into how CAF programs shape the immune landscape. Favorable prognostic groups defined by CAF signatures were consistently associated with lymphocyte-rich, immune-active microenvironments, whereas poor prognostic groups exhibited myeloid-dominant and inflammation-associated immune profiles. These findings support a model in which inflammatory CAFs contribute not only to tumor-promoting inflammation but also to immune exclusion and suppression, potentially through chemokine-mediated myeloid recruitment and TGF-β-associated stromal remodeling [11,21,23,27,28]. The strong concordance between CAF-derived risk groups and immune activation states suggests that CAFs act as upstream regulators of immune contexture in HNSCC.

Several recent studies have reported the prognostic significance of CAF heterogeneity in HNSCC using integrated single-cell and bulk transcriptomic analyses [29,30,31,32,33]. These studies consistently demonstrate that specific CAF phenotypes, particularly inflammatory and myofibroblastic subsets, are associated with tumor aggressiveness, immune modulation, and patient outcomes. Building on this growing body of literature, we constructed CAF subset-specific risk signatures using LASSO regression and subsequently integrated these signatures using recursive partitioning analysis to define CAF-driven patient subgroups with distinct overall survival, which were further validated in an independent cohort. These findings extend previous CAF-focused studies by linking CAF heterogeneity to integrated patient-level prognostic stratification and by demonstrating the translational relevance of CAF transcriptional programs in HNSCC.

The enrichment of immune checkpoint and effector gene expression in favorable prognostic nodes further raises the possibility that CAF-defined stromal states may influence responsiveness to immunotherapy. Given that immune checkpoint inhibitors have become a cornerstone of HNSCC treatment, CAF-based stratification could provide complementary information beyond tumor-intrinsic biomarkers, such as PD-L1 expression [5,27,34,35,36]. In this context, inflammatory CAF programs may represent actionable components of the tumor microenvironment, either as therapeutic targets themselves or as modulators of immunotherapeutic efficacy.

Our bulk RNA sequencing analysis of primary CAFs provides additional biological validation by demonstrating that myCAF-like and iCAF-like transcriptional programs are maintained ex vivo. This finding is consistent with previous studies showing that distinct CAF phenotypes can persist beyond the immediate tumor microenvironment and retain characteristic transcriptional states in culture [20,24]. Together, these observations suggest that CAF phenotypes identified by scRNA-seq reflect relatively stable cellular states rather than transient transcriptional responses to local cues, thereby reinforcing their relevance as biologically meaningful and functionally distinct stromal entities. Notably, myCAF-like and iCAF-like primary CAFs showed distinct anatomical origins, with myCAF-like samples derived exclusively from hypopharyngeal cancers and most iCAF-like samples from oropharyngeal cancers. Although limited by sample size, this observation aligns with our single-cell findings and suggests that anatomical site-specific contexts may influence CAF transcriptional programs in HNSCC.

Several limitations merit consideration. First, given the marked heterogeneity of HNSCC with respect to anatomical site, HPV status, treatment background, and tumor microenvironmental context, CAF transcriptional programs and their prognostic impact should be interpreted as context-dependent rather than uniform across all cases. Second, although our integrative approach leverages multiple independent datasets, functional validation of CAF–immune interactions was not directly addressed. Third, spatial relationships between CAF subsets and immune cells could not be resolved using bulk or dissociated single-cell approaches. Future studies employing spatial transcriptomics and functional perturbation models will be essential to clarify the mechanistic basis of CAF-driven immune modulation.

## 5. Conclusions

In summary, this study demonstrates that CAF heterogeneity—particularly inflammatory CAF programs—defines clinically meaningful stromal states that shape immune landscapes and patient survival in HNSCC. By moving beyond descriptive classification, our findings position CAF-centered transcriptional programs as integrative biomarkers and potential therapeutic targets within the tumor microenvironment.

## Figures and Tables

**Table 1:** Relationship between CAF risk scores (OS) and clinical parameters in 520 patients with HNSCC.

Variables	myCAF (OS)	iCAF1 (OS)	iCAF2 (OS)	apCAF (OS)	ecmCAF (OS)
	Mean (SE)	Mean (SE)	Mean (SE)	Mean (SE)	Mean (SE)
HPV status					
Negative	1.349 (0.053)	1.258 (0.035)	1.260 (0.036)	1.286 (0.038)	1.194 (0.027)
Positive	0.850 (0.088)	0.851 (0.071)	0.803 (0.066)	0.794 (0.064)	0.815 (0.054)
*p*-value	<0.0001	<0.0001	<0.0001	<0.0001	<0.0001
Primary lesion					
Hypopharynx	2.336 (0.591)	1.353 (0.275)	1.757 (0.424)	1.501 (0.240)	1.180 (0.185)
Larynx	1.137 (0.072)	1.034 (0.055)	1.139 (0.057)	1.138 (0.060)	0.978 (0.037)
Oral cavity	1.336 (0.061)	1.287 (0.041)	1.221 (0.040)	1.275 (0.044)	1.223 (0.032)
Oropharynx	0.685 (0.098)	0.676 (0.079)	0.759 (0.117)	0.606 (0.079)	0.683 (0.055)
*p*-value	<0.0001	<0.0001	0.0001	<0.0001	<0.0001
T factor					
T1–2	1.081 (0.055)	1.080 (0.047)	1.045 (0.048)	0.991 (0.041)	1.023 (0.037)
T3–4	1.374 (0.069)	1.250 (0.043)	1.262 (0.043)	1.330 (0.049)	1.191 (0.033)
*p*-value	0.001	0.008	0.0009	<0.0001	0.0008
N factor					
Negative	1.228 (0.088)	1.169 (0.052)	1.114 (0.049)	1.164 (0.060)	1.102 (0.037)
Positive	1.303 (0.056)	1.210 (0.044)	1.248 (0.046)	1.242 (0.044)	1.157 (0.036)
*p*-value	0.472	0.542	0.048	0.292	0.282
M factor					
M0	1.247 (0.048)	1.171 (0.032)	1.161 (0.033)	1.184 (0.035)	1.126 (0.026)
M1	1.657 (0.424)	1.786 (0.504)	1.747 (0.428)	1.703 (0.224)	1.115 (0.237)
*p*-value	0.389	0.289	0.243	0.081	0.967
TNM stage					
I–II	1.068 (0.066)	1.074 (0.056)	1.027 (0.052)	0.999 (0.050)	1.053 (0.048)
III–IV	1.308 (0.057)	1.212 (0.038)	1.215 (0.039)	1.247 (0.041)	1.143 (0.029)
*p*-value	0.006	0.042	0.004	0.0002	0.112

CAF, cancer-associated fibroblast; HNSCC, head and neck squamous cell carcinoma; HPV, human papillomavirus; OS, overall survival; SE, standard error.

**Table 2:** Univariate and multivariate survival analyses of OS and PFS in 520 patients with HNSCC.

Variables	Overall Survival (OS)			Progression-Free Survival (PFS)		
	Univariate	Multivariate		Univariate	Multivariate	
	*p*-Value	HR (95% CI)	*p*-Value	*p*-Value	HR (95% CI)	*p*-Value
HPV status (ref: negative)						
Positive	0.137			0.050		
Primary lesion (ref: Oropharynx)						
Hypopharynx	0.058	2.117 (0.511–8.772)	0.301	0.057		
Larynx	0.127	1.100 (0.451–2.683)	0.833	0.542		
Oral cavity	0.045	1.443 (0.614–3.387)	0.400	0.149		
T factor (ref: T1–2)						
T3–4	0.0002	1.833 (1.103–3.047)	0.019	0.001	1.271 (0.769–2.099)	0.350
N factor (ref: negative)						
Positive	0.037	1.193 (0.814–1.750)	0.366	0.063		
M factor (ref: M0)						
M1	0.003	4.322 (1.457–12.826)	0.008	0.284		
TNM stage (ref: I–II)						
III-IV	0.010	0.787 (0.399–1.553)	0.489	0.011	1.695 (0.886–3.242)	0.111
myCAF risk score (ref: low)						
High	<0.0001	1.684 (1.153–2.458)	0.007	<0.0001	1.012 (0.657–1.559)	0.957
iCAF1 risk score (ref: low)						
High	<0.0001	1.625 (1.135–2.327)	0.008	<0.0001	1.844 (1.205–2.823)	0.005
iCAF2 risk score (ref: low)						
High	<0.0001	1.581 (1.095–2.283)	0.015	<0.0001	2.395 (1.534–3.740)	0.000
apCAF risk score (ref: low)						
High	<0.0001	1.206 (0.804–1.809)	0.366	<0.0001	1.640 (1.107–2.431)	0.014
ecmCAF risk score (ref: low)						
High	<0.0001	1.183 (0.820–1.707)	0.368	<0.0001	1.394 (0.920–2.114)	0.118

## Data Availability

The original data presented in this study are openly available in GSE164690, https://www.ncbi.nlm.nih.gov/geo/query/acc.cgi?acc=GSE164690 (accessed on 1 June 2023); GSE65858, https://www.ncbi.nlm.nih.gov/geo/query/acc.cgi?acc=GSE65858 (accessed on 30 April 2024); and on the FireBrowse website, http://firebrowse.org/ (accessed on 6 May 2020).

## Supplementary Materials

The following supporting information can be downloaded at https://www.mdpi.com/article/10.3390/cancers18020215/s1, Figure S1: Feature selection for CAF subset-specific prognostic models, Figure S2: Recursive partitioning analysis for PFS, Figure S3: Validation of immune landscapes and pathways in the GSE65858 cohort; Table S1: Relationship between CAF risk scores (PFS) and clinical parameters in 520 patients with HNSCC.
