# Supplementary material for: Cancer-Associated Fibroblast Heterogeneity Shapes Prognosis and Immune Landscapes in Head and Neck Squamous Cell Carcinoma
Source: Cancers (Basel). 2026 Jan 9;18(2):215. doi: 10.3390/cancers18020215 (PMC12838901; doi:10.3390/cancers18020215)
Supplement: Supplementary file 1 [file cancers-18-00215-s001.zip › cancers-4074840-supplementary.pdf]

Supplementary Figure 1

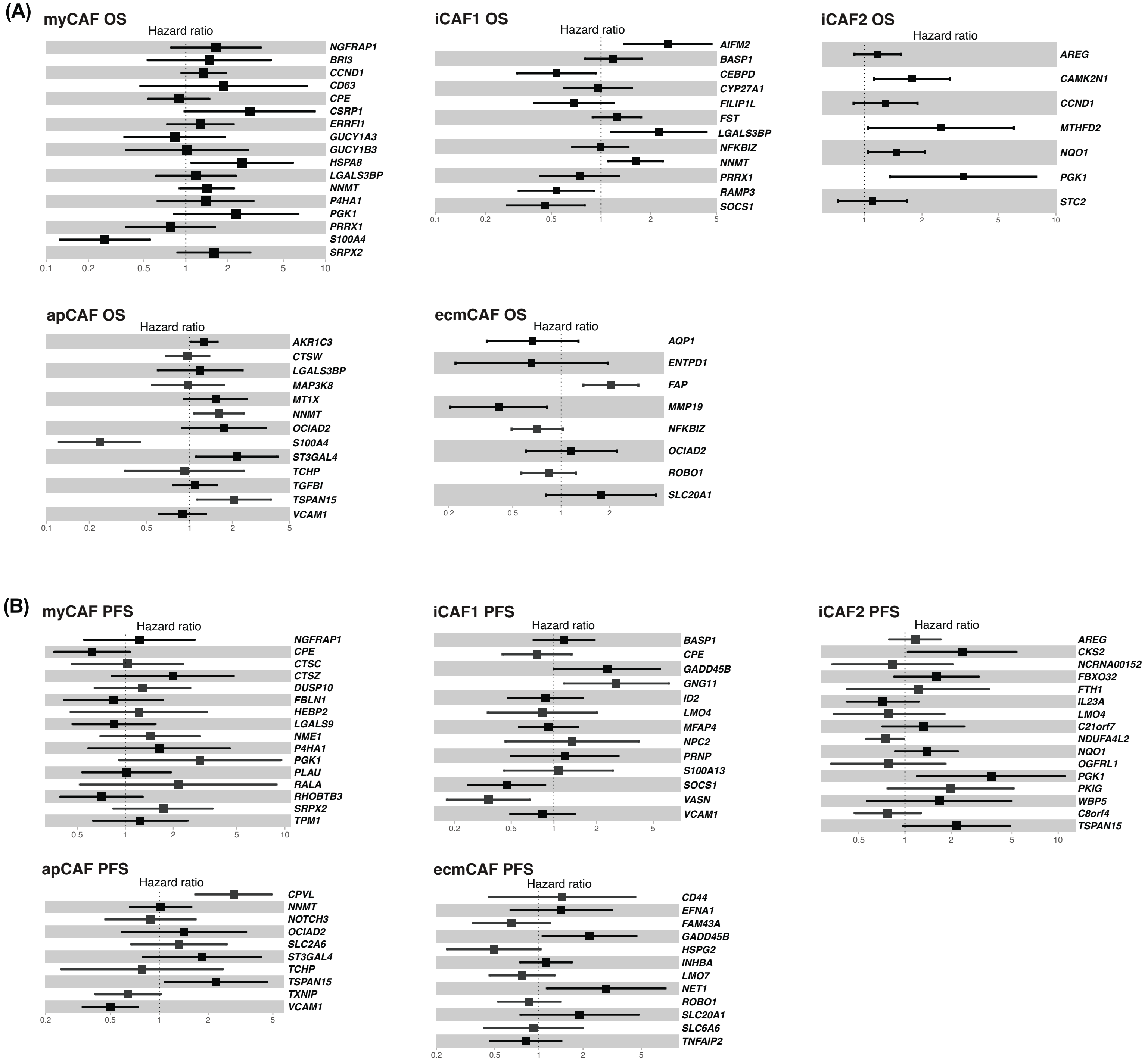

Figure S1. Feature selection for CAF subset–specific prognostic models.
(A) Forest plots showing hazard ratios and 95% confidence intervals (CIs) of least absolute shrinkage and selection operator (LASSO)-selected DEGs for OS in each CAF subset.
(B) Forest plots showing hazard ratios and 95% CIs of LASSO-selected DEGs for PFS in each CAF subset.

Supplementary Figure 2

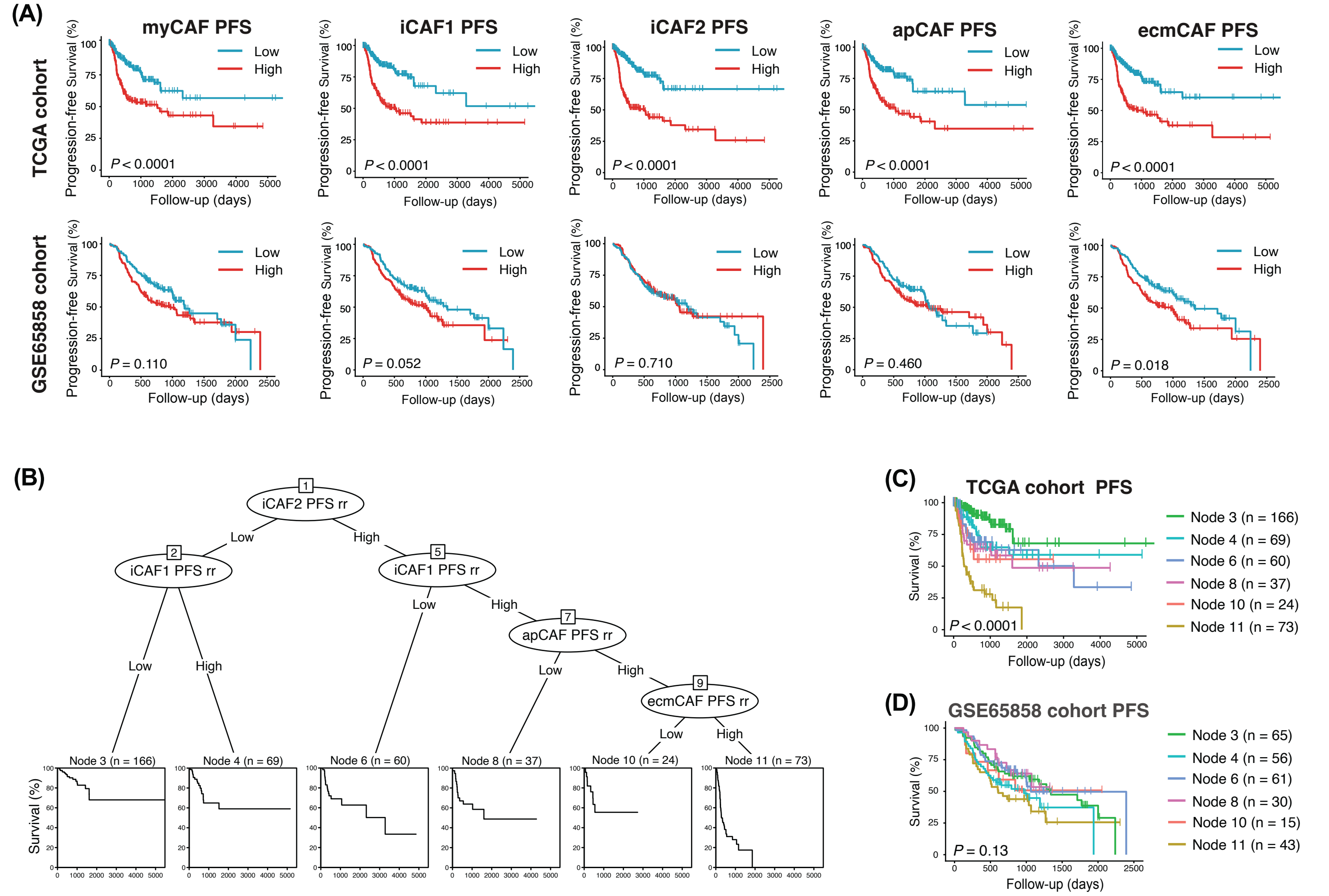

Figure S2. Recursive partitioning analysis for PFS.
(A) Kaplan–Meier curves for PFS based on CAF risk scores in the TCGA and GSE65858 cohorts.
(B) Conditional inference tree for PFS generated in the TCGA cohort.
(C) Kaplan–Meier curves for PFS across terminal nodes in the TCGA cohort.
(D) Kaplan–Meier curves for PFS across terminal nodes in the GSE65858 cohort.

Supplementary Figure 3

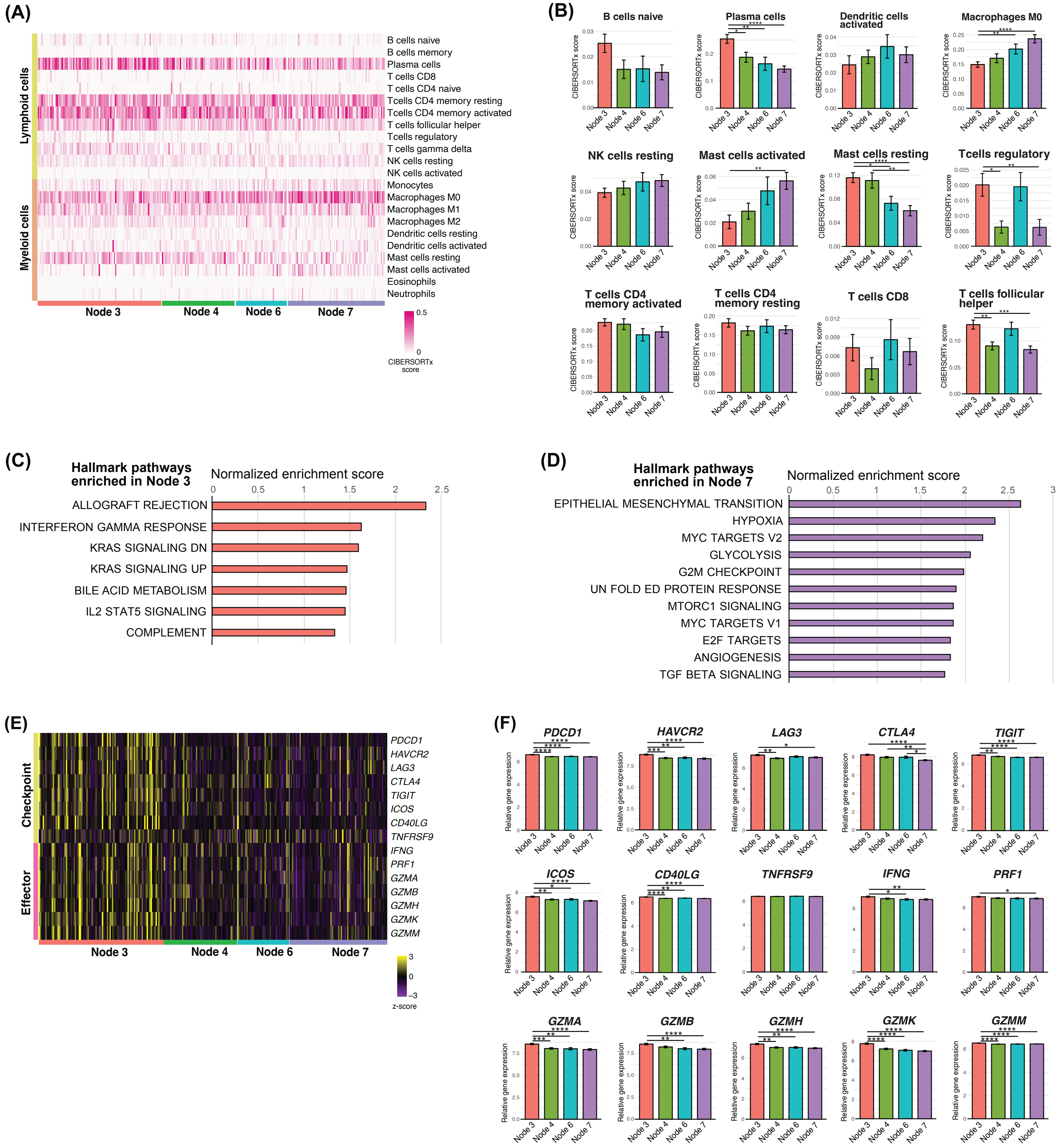

Figure S3. Validation of immune landscapes and pathways in the GSE65858 cohort.
(A) Heatmap showing immune cell abundance estimated using CIBERSORTx.
(B) Bar graphs displaying immune cell abundance scores across terminal nodes.
(C–D) GSEA showing pathways enriched in Node 3 and Node 7.
(E) Heatmap showing expression of immune effector and immune checkpoint–related genes.
(F) Bar graphs displaying relative gene expression levels in terminal nodes.

Table S1. Relationship between CAF risk scores (PFS) and clinical parameters in 520 patients with HNSCC

Variables	myCAF (PFS)	iCAF1 (PFS)	iCAF2 (PFS)	apCAF (PFS)	ecmCAF (PFS)
	Mean (SE)	Mean (SE)	Mean (SE)	Mean (SE)	Mean (SE)
HPV status					
Negative	1.323 (0.050)	1.364 (0.085)	1.430 (0.066)	1.329 (0.057)	1.264 (0.042)
Positive	0.775 (0.067)	0.829 (0.087)	0.858 (0.089)	0.795 (0.066)	0.821 (0.050)
P-value	< 0.0001	< 0.0001	< 0.0001	< 0.0001	< 0.0001
Primary lesion					
Hypopharynx	1.654 (0.318)	1.581 (0.324)	1.327 (0.209)	1.637 (0.363)	1.933 (0.378)
Larynx	1.010 (0.059)	0.953 (0.072)	0.998 (0.087)	1.087 (0.075)	0.941 (0.054)
Oral cavity	1.339 (0.057)	1.428 (0.100)	1.495 (0.075)	1.327 (0.065)	1.286 (0.046)
Oropharynx	0.694 (0.087)	0.642 (0.070)	0.742 (0.115)	0.706 (0.071)	0.760 (0.078)
P-value	< 0.0001	0.002	< 0.0001	0.001	< 0.0001
T factor					
T1-2	1.134 (0.063)	1.151 (0.060)	1.263 (0.094)	1.162 (0.079)	1.071 (0.053)
T3-4	1.281 (0.059)	1.343 (0.116)	1.364 (0.071)	1.275 (0.062)	1.259 (0.048)
P-value	0.091	0.1408	0.39	0.259	0.009
N factor					
Negative	1.136 (0.070)	1.351 (0.161)	1.192 (0.078)	1.147 (0.072)	1.110 (0.050)
Positive	1.287 (0.058)	1.209 (0.049)	1.419 (0.075)	1.240 (0.057)	1.225 (0.051)
P-value	0.097	0.400	0.037	0.312	0.106
M factor					
M0	1.215 (0.044)	1.252 (0.073)	1.317 (0.058)	1.217 (0.049)	1.171 (0.036)
M1	1.960 (1.026)	1.470 (0.654)	1.872 (1.031)	2.038 (1.270)	1.274 (0.618)
P-value	0.600	0.795	0.686	0.634	0.895
TNM stage					
I-II	1.078 (0.072)	1.181 (0.077)	1.156 (0.086)	1.096 (0.088)	1.078 (0.071)
III-IV	1.259 (0.052)	1.285 (0.089)	1.368 (0.069)	1.264 (0.057)	1.208 (0.041)
P-value	0.043	0.379	0.056	0.111	0.118

CAF, cancer-associated fibroblast; HNSCC, head and neck squamous cell carcinoma; HPV; PFS, progression-free survival; human papillomaviru; SE, standard error.
